# Descriptive data on cardiovascular and metabolic risk factors in ambulatory and non-ambulatory adults with cerebral palsy

**DOI:** 10.1016/j.dib.2015.10.045

**Published:** 2015-11-10

**Authors:** P.G. McPhee, J.W. Gorter, L.M. Cotie, B.W. Timmons, T. Bentley, M.J. MacDonald

**Affiliations:** a Department of Kinesiology, McMaster University, 1280 Main St. W., Hamilton, ON, Canada L8S 4K1; b CanChild Centre for Childhood Disability Research, Department of Pediatrics, McMaster University, 1400 Main St. W., Hamilton, ON, Canada L8S 1C7; c Child Health & Exercise Medicine Program, Department of Pediatrics, McMaster University, 1400 Main St. W., Hamilton, ON, Canada L8S 1C7; d Department of Medicine, Division of Physical Medicine and Rehabilitation, McMaster University, 1280 Main St. W., Hamilton, ON, Canada L8S 4K1

**Keywords:** Obesity, Hypertension, Cerebral palsy, Adult, Cardiometabolic health

## Abstract

Forty-two participants with cerebral palsy were recruited for a study examining traditional and novel indicators of cardiovascular risk (McPhee et al., 2015 [1]). Data pertaining to the prevalence of obesity, smoking, hypertension, and metabolic risk are provided. These data are presented along with the scoring methods used in evaluation of the study participants. Percentages are included for comparative purposes with the existing literature.

**Specifications table**

**Table:** 

Subject area	*Biology*
More specific subject area	*Obesity, cardiovascular and metabolic risk*
Type of data	*Table*
How data were acquired	*Clinical evaluations; Blood pressure via automated sphygmomanometer (Dinamap Pro 100, Critikon LCC, Tampa, Fla, USA); Full lipid panel (Hamilton Regional Laboratory Medicine Program – McMaster Medical Centre Campus); Glucose (Glucose Hexokinase Reagent Set, Pointe Scientific, Inc. Canton, MI, USA).*
Data format	*Raw, Analyzed*
Experimental factors	*Participants provided a 12hr fasting venous blood sample for serum analysis*
Experimental features	*Prevalence of cardiovascular disease risk was obtained using criteria from the Framingham Risk Score. Obesity and metabolic risk were determined using values from the literature.*
Data source location	*Hamilton, Ontario, Canada*
Data accessibility	*Data are with this article*

**Value of the data**

• There is a paucity of literature reporting the prevalence of cardiovascular disease risk in adults with cerebral palsy; a population believed to be at elevated risk due to their mobility impairments.
• These data are included for comparative purposes of the existing literature, and may inform systematic and/or meta-analytic reviews.
• These data may inform future studies in non-ambulatory adults with cerebral palsy as they presented greater absolute risk for cardiovascular disease.

## 1. Data, experimental design, materials and methods

### 1.1 Data

In the present data, we highlight the prevalence of cardiovascular and metabolic risk in adults with cerebral palsy (CP) who are either ambulatory or non-ambulatory. We provide cardiovascular disease (CVD) risk factor information that is consistent with predictors from the Framingham Heart Study [2]. We also present individuals who are at elevated risk for CVD based on obesity, and lipid and glucose levels.

### 1.2 Subjects

Study participants’ characteristics are described elsewhere [1].

### 1.3 Methods

Measurements of supine brachial artery systolic blood pressure, diastolic blood pressure, and mean arterial pressure were obtained using an automated sphygmomanometer (Dinamap Pro 100, Critikon LCC, Tampa, Fla, USA) in all 42 participants. Hypertension was defined as per the Eighth Joint National Committee [3].

Supine height without shoes was measured to the nearest 0.5 cm using an anthropometric tape measure. Body mass was measured to the nearest 0.1 kg using a digital wheelchair scale (Detecto Scales, FHD Series, Webb City, Missouri, USA). Body mass index (BMI) was determined by dividing the participant׳s weight (kg) by their height squared (m^2^). Waist circumference was measured while in a supine position using an adapted technique as per the National Institutes of Health (NIH) [4]. All anthropometric measures were collected in all 42 participants.

Participants provided a 12 h fasting venous blood sample for serum analysis. Thirty-three of the 42 participants provided a venous blood sample. Blood draws were not acquired on one ambulatory and eight non-ambulatory participants due to contractures, spasticity, or insufficient vein size. The samples were analyzed for lipids; total cholesterol (TC), high-density lipoprotein cholesterol (HDL-C), low-density lipoprotein cholesterol (LDL-C), TC/HDL-C, and triglycerides. Participant labeled Eppendorf tubes were sent to Hamilton Regional Laboratory Medicine Program – McMaster Medical Centre Campus for analysis of the previously identified lipid markers. Glucose was examined using the Glucose Hexokinase Reagent Set (Pointe Scientific, Inc. Canton, MI, USA). All coefficients of variation between duplicates were <10% for each metabolic marker.

Descriptive results of lifestyle-related and metabolic risk factors are shown in Table 1. Clinical cholesterol levels for CVD risk were >5.20 mmol/L for TC; <1.04 mmol/L for HDL-C; >3.40 mmol/L for LDL-C; >6.0 for TC/HDL-C as per Canadian Cardiovascular Society guidelines [5]. Central obesity was defined as having a waist circumference ≥102 cm for men or ≥88 cm for women as per the NIH [4]. A BMI ≥30 kg/m^2^ was defined as being obese as per the NIH [4]. Hyperglycemia was defined as ≥100 mg/dL as per American Diabetes Association [6].

## Figures and Tables

**Table 1:** Prevalence of obesity and cardiovascular and metabolic risk factors among participants.

Risk factor	Total (*n*=42)	Ambulatory (*n*=14)	Non-ambulatory (*n*=28)
BMI≥30 kg/m^2^	9 (21.4)	3 (21.4)	6 (21.4)
Central obesity	12 (28.6)	4 (28.6)	8 (28.6)
Hypertensive BP	6 (14.3)	0	6 (21.4)
Hypercholesterolemia	13 (39.4)	5 (38.5)	8 (40.0)
Smoker	3 (7.14)	2 (14.3)	1 (3.57)
Low HDL-C	8 (24.2)	2 (15.4)	6 (30.0)
High LDL-C	13 (39.4)	6 (46.2)	7 (35.0)
TC/HDL-C	4 (12.1)	1 (7.7)	3 (15.0)
Hypertriglyceridemia	8 (24.2)	2 (15.4)	6 (30.0)
Hyperglycemia	17 (51.5)	6 (46.1)	11 (55.0)

Values are *n* (%).

All metabolic markers (ambulatory *n*=13; non-ambulatory *n*=20).
